# Appendicitis*Clinical lecture delivered at the Rooesvelt Hospital, Nov. 10, 1894.

**Published:** 1895-01

**Authors:** Charles McBurney

**Affiliations:** New York, Professor of Surgery in the College of Physicians and Surgeons


### APPENDICITIS.*

By CHARLES McBURNEY, M. D., New York,

Professor of Surgery in the College of Physicians and Surgeons.

This young man is nineteen years old. He was well until last Tuesday (Nov. 6th), when he began to complain of pain in the lower part of the abdomen, which lasted for about twenty-four hours and then localized itself in the right iliac fossa. The general distribution of the pain during the early stage of appendicitis is one of the reasons why this disease is so often overlooked. The physician is called in and finds the patient complaining of general abdominal pain, as in an attack of ordinary colic; the patient is not examined for localized tenderness. A hypodermic injection of morphine is perhaps given; this obscures the symptoms and leads to further delay. After the lapse of some hours the pain returns, and the injection is repeated. Meanwhile, the disease is progressing. The temperature and pulse, during this period, may not be above the normal.

On the first day this patient was taken ill, he thought he had a "stomach ache." The pain was in the lower part of the abdomen, and there was some vomiting. On the second day the pain was localized in the right iliac fossa and the vomiting had ceased. On the third day the pain became so severe that he called in a physician. His temperature on that day was 103.5 degrees F., pulse 120. A diagnosis of acute appendicitis was at once made and the patient sent to the hospital for operation. Numerous directions have been laid down as to the proper time to operate in these cases. Some claim that we should wait until a perceptible tumor can be made out; others that we should wait until a given day, the fourth or sixth day, or even later, when it is stated that the operation is comparatively easy and free from danger. These doctrines are absolutely wrong. When we have to deal with a disease which in its full development threatens the life of the patient, the time to operate is before that full development is reached, providing there is no special danger in doing the operation at an earlier period, and no special advantage in deferring it until a later one.

*Clinical lecture delivered at the Rooesvelt Hospital, Nov. 10, 1894.

From the history of this case, I feel quite certain that perforation of the appendix has occurred, and that the infection coming out of this opening has set up a suppurative process within the peritoneum which is now spreading. When we come to examine this patient we find that the abdomen is somewhat distended. There is a limited area of tenderness between the right anterior iliac spine and the umbilicus, and the percussion note is tympanitic over this entire region. No tumor can be made out. It is exceedingly common, in these cases, to get tympanitic percussion, for the reason that the process is an intra-peritoneal one, and the collection of pus lies between the folds of intestines. You may have a pint of pus concealed deep down in the belly, and over this you may have the distended intestines which gave rise to the tympanitic percussion. In this case, the collection of pus surrounding the perforated appendix is probably bound in by adhesive material; if this be left to itself, a number of different things might happen—the collection of pus might increase in quantity, break down the inflammatory wall which surrounds it, and distribute itself throughout the entire peritoneal cavity; this would produce collapse and probably result in death. Or, if the inflammatory wall surrounding the collection of pus does not give way, the pus may in the course of a few days find its way into the intestines by ulceration; if that occurs, the patient's pain and fever will probably disappear, but the result is unfortunate; the collapsed pus sack remains lined by septic material and is liable to refill. Or the process may advance anteriorly, and the pus force its way out through the abdominal wall. Or it may burrow among the folds of intestine and force its way through the rectum or colon, or up to the kidney. I saw one case in which it passed through the diaphragm into the lung, and the patient expectorated the contents of the abscess.

From the situation of the tenderness in this case, I judge that the appendix lies somewhat in this position, that is, on the outer side of the caput coli. The method of approaching a collection of pus like this is important. If we opened into this abdomen in the middle line, we would have great difficulty in reaching the lesion, and the pus would probably distribute itself all over the intestines and the case end fatally.

We want to get in between the caput coli and the outer wall. I shall therefore make my incision in that location and allow the pus to pass out slowly, gradually enlarging the opening, if necessary. If the appendix can be taken away readily, I shall do so. If, however, it is concealed or very difficult to remove, I shall not endanger the patient by making a prolonged effort to break down the adhesions, but will leave the wound open, and trust that the remainder of the appendix will slough off. In order to ascertain whether pus exists or not, in a case like this, exploratory puncture was formerly advocated by some surgeons, and sometimes this advice is given even to-day. There are good reasons why this procedure should not be employed. Suppose you thrust a needle in here, where the percussion is tympanitic and where we have reaon to believe that the intestines lie in front of the abscess. Such a puncture will involve the intestines, and this will handicap you in your work; the gut must then be sewn up, or else you will see fæcal material squirt out through the openings. If the patient is sufficiently ill to require operation, you should be able to make your diagnosis without the aid of the needle. It is not necessary to find out whether pus is there; that question can be left out entirely. Furthermore, during the withdrawal of your needle after exploratory puncture, you are liable to infect all the tissues through which it passes after leaving the pus sac.

I shall now make my incision, first cutting through the external oblique aponeurosis, and then through the internal oblique and transverse muscles. Before opening the peritoneum, it is well to check all hemorrhage, so that the blood will not interfere with the deeper work. I have now made a small opening into the peritoneum, through which the pus is escaping; this is immediately mopped up with sponges, so as to prevent it from infecting the neighboring tissues. The opening in the peritoneum is gradually increased in size by loosening the adhesions. Here is the appendix which is readily detached and removed. I shall not close the wound, but treat it by the open method, so as to allow the pus a ready means of escape externally. After cleansing the abscess cavity by gently mopping it out, I pack the entire cavity with iodoform gauze, which will drain away all fluid much better than any tube. The dressing and packing will be changed in three days.

